# Pattern of varicocele vein blood gases in patients undergoing microsurgical Varicocelectomy

**DOI:** 10.1186/s12894-018-0411-y

**Published:** 2018-11-13

**Authors:** Khaleeq ur Rehman, Hafsa Zaneb, Abdul Basit Qureshi, Ahsan Numan, Muhammad Shahbaz Yousaf, Imtiaz Rabbani, Habib Rehman

**Affiliations:** 1 Department of Urology & Andrology, FMH College of Medicine & Dentistry, Shadman, Lahore, Pakistan; 2 Department of Anatomy and Histology, University of Veterinary and Animal Sciences, Lahore, Pakistan; 3 Department of Surgery, Services Institute of Medical Sciences, Lahore, Pakistan; 4 Department of Neurology, Services Institute of Medical Sciences, Lahore, Pakistan; 5 Department of Physiology, University of Veterinary and Animal Sciences, Lahore, Pakistan

**Keywords:** Venous blood gases, Varicocele, Scrotal Doppler ultrasonography, Infertility, Testicular blood flow

## Abstract

**Background:**

Varicocele is known to be associated with infertility and sperm disorders. The exact cause of this ailment is not fully understood. There are limited numbers of studies where venous blood gases (VBGs) of varicocele veins were determined with conflicting results. Therefore, we have investigated the pattern of VBGs in both internal spermatic and external spermatic varicocele veins and correlation with semen quality parameters in infertile individuals who underwent left microsurgical varicocelectomy.

**Methods:**

Patients (*n* = 27) undergoing left microsurgical varicocelectomy at a tertiary care hospital, were included in the study. Before surgery, semen parameters and scrotal color Doppler ultrasonography was performed. During surgery, blood sample was drawn from varicocele veins (internal spermatic and external spermatic veins) and a peripheral arm vein of the same patient as a control. The VBGs of all veins under study were estimated and compared with each other. The VBGs were also correlated with various semen quality parameters. Data, expressed as Mean ± SD, regarding VBGs in three veins were analyzed using one-way ANOVA. The correlation between VBGs and semen quality parameters was determined using Pearson’s correlation. Differences were considered significant at *p* < 0.05.

**Results:**

The pH was found to be higher (*p < 0.01*) in the internal spermatic vein compared with the external spermatic and the peripheral veins. Partial pressure of oxygen (pO_2_) and oxygen saturation (sO_2_) were higher (*p* < 0.01) in the internal spermatic vein compared with the peripheral vein. However, concentration of bicarbonate (HCO_3)_ was lower (*p* < 0.01) in both veins compared with the peripheral vein. Partial pressure of carbon dioxide (pCO_2_) was also lower (*p < 0.01*) in the varicocele veins compared with the control vein.

**Conclusion:**

The internal spermatic veins had higher pH and oxygen tension, but lower HCO_3_ and pCO_2_ levels compared with the control peripheral veins. External spermatic veins had lower pCO_2_ and HCO_3_ but other VBGs were similar to the peripheral veins. The shift of VBGs of internal spermatic vein toward arterial blood pattern may be a missing link to understand the pathophysiology of varicocele.

## Background

Varicocele is known to be associated with infertility and sperm disorders. Most researchers believe that renospermatic reflux is the main mechanism behind all mal-effects of varicocele [1–6]. There is a possibility that varicocele has various types that exert influence through different mechanisms. We have already reported an interesting example of varicocele that appeared in 23% hypogonadotropic hypogonadism patients after treatment with gonadotropins [7]. The variable behaviour of varicocele on spermatogenesis is intriguing and far from being understood. Why some individuals with varicocele develop sperm disorders but others don’t is not clear. It appears that some other mechanisms coexist. A previous experimental study described the presence of an arteriovenous communications between testicular artery and adjacent veins [8]. In spermatic cord of bull, when gas was pushed into the testicular venous system, it could be recovered from the arterial side [9]. Arteriovenous anastomosis has been reported in human spermatic cord as well [10]. It was reported that a periarterial capillary network exists that connects the human testicular artery with adjacent pampiniform plexus [11–13]. These findings have not been given due importance. Based on the animal experimental studies mentioned, we presumed that these types of communications might be present in the human varicocele veins and subsequently may change VBG patterns of varicocele veins. There are only a limited numbers of studies where VBGs of the varicocele veins of testes were determined. These studies had unfortunately conflicting results [14–16]. To the best of our knowledge, there is no study that describes the VBGs profile of both internal and external spermatic varicocele veins in a single subject and its relation with semen quality. Therefore, the present study aimed at investigating the profile of VBGs in the varicocele veins (internal spermatic and external spermatic veins) of infertile individuals and to compare these variables with peripheral vein blood from the same individual undergoing left microsurgical varicocelectomy. Attempts were also carried out to explore a possible correlation between various variables of VBGs and semen quality parameters like semen volume, appearance, sperm count, progressive and non-progressive sperm motility and sperm morphology.

## Methods

### Subjects

Twenty-seven consecutive patients diagnosed with infertility and varicocele, undergoing microsurgical varicocelectomy under spinal anaesthesia, were included in the study after informed written consent. The calculated sample size was 23 using the following equation:

N = [(Z_α_ + Z_β_)/C]^2^ + 3. The values for α (two tailed), β and r were 0.050, 0.300 and 0.500 respectively.

The study was approved by the Institutional Review Board of Fatima Memorial Hospital, College of Medicine & Dentistry Lahore-Pakistan.

### Study design

Twenty to 45 years-old individuals with Grade 2 or 3 varicocele [17] having varicocele vein diameter of > 2.5 mm on scrotal color Doppler ultrasonography (CDUS) [18] with at least 1-year of infertility were included in the study. Semen analysis was carried out at least twice to confirm semen disorders. Patients with any chronic illness (hepatitis C or B, cardiac or pulmonary disorders), on prolonged drugs having side effects for spermatogenesis (e.g., antiviral drugs, chemotherapeutic agents), hormonal disorders (hypogonadism, hypothyroidism etc.), on prolonged antidepressants or drugs addicts were excluded from study. Patients with male or female sexual dysfunction leading to decreased frequency of intercourse (less than twice per week) and patients with significant female factor infertility were also excluded from the study.

### Semen collection and analysis

Semen samples were collected at 3–4 days of abstinence and were processed for determination of semen quality parameters (semen volume, appearance, sperm count, progressive and non-progressive motility, morphology and other microscopic details) as suggested by the World Health Organization [19].

### Scrotal ultrasonography for varicocele and scrotal contents

Scrotal CDUS was performed (Voluson General Electronics 30) using 10 mHz linear probe [20]. Briefly, grey scale ultrasonography was done to detect any other associated abnormality. Testicular and epididymal diameters were noted. Using CDUS, peak systolic velocities (PSVs) and resistive indices (RIs) of the subcapsular and intraparenchymal branches of testicular artery were determined. Diameter and backflow status of varicocele veins was recorded in lying and standing position. Subinguinal microsurgical varicocelectomy was performed as per standard practice [20, 21] under spinal anaesthesia.

### Acquisition of blood gases

During surgery just before ligation of varicocele veins, 2.0 mL blood was drawn with a 27G needle inserted towards the direction of testes, in a heparinized syringe, from 1 to 2 internal spermatic veins and from external spermatic vein if found dilated (> 2.5 mm diameter). Peripheral blood sample from same individual was also drawn simultaneously from the wrist vein as a control. The patients did not receive oxygen inhalation at the time of blood sampling as well as for the previous 15 min at minimum. All patients maintained 97% or more oxygen saturation at room air. The blood samples were taken free of air, sealed, and blood gas analysis was performed immediately with a blood-gas analyser (Cobas b 121–Hoffmann La Roche, Inc., Germany). The pH, partial pressure of oxygen (pO_2_), partial pressure of carbon dioxide (pCO_2_), oxygen saturation (sO_2_), and bicarbonate (HCO_3_) values were determined.

### Statistical methods

Data are represented as mean ± standard deviation. Means values of VBGs in all 3 veins were compared with one-way analysis of variance using SPSS software (SPSS Inc., USA). Tukey’s post hoc test was carried out to identify individual differences. The Pearson correlation test was employed to determine correlations between different variables. Level of significance was set at *p* < 0.05.

## Results

Out of twenty-seven patients, twenty-three (85.18%) had G2 varicocele and 4 (14.8%) had G3 varicocele. Age of the patients ranged from 20 to 43 (29.38 ± 7.94) years. Ten (37.04%) patients were 20–30 years old, 13 (48.15%) were between 31 to 40 years and 4 (14.81%) patients were 41–43 years old. Most of them (81.48%) were non-smokers.

### Baseline semen and scrotal CDUS findings

The mean diameters of the varicocele veins of the patients were 3.40 ± 0.86 mm and 3.74 ± 0.86 mm at lying and standing positions, respectively. The PSV and RI of subcapsular and intraparenchymal artery along with testicular volume are summarized in Table 1. The semen quality parameters in terms of sperm count, progressive motility, non-progressive motility, immotililty, and morphology of spermatozoa of the studied individuals were 33.45 ± 27.78 × 10^6^ per mL, 16.89 ± 14.4%, 17.59 ± 13.49%, 58.11 ± 27.43%, and 7.11 ± 7.00% respectively (Table 1). Among semen parameters, sperm morphology had significant correlation (*r* = 0.463; *p* < 0.05) with PSV of subcapsular artery of left testes, testicular volume (*p* < 0.05, *r* = 0.407) and transverse diameter of testes. (*p* < 0.05; *r* = 0.439). Sperm count had significant negative correlation (*r* = − 0.76; *p* < 0.05) with RI of left intraparenchymal artery, whereas progressive motility of sperms had negative correlation (*r* = − 0.498; *p* < 0 .01) with RI of left intraparenchymal artery as well as RI of left subcapsular artery (*r* = − 0.505; *p* < 0.01) (Table 2). On comparison between varicocele diameter and semen parameters, no correlations were appreciated.

**Table 1:** Baseline characteristics of various parameters of varicocele patients (*n* = 27)

Variables	Mean	±SD	Minimum	Maximum
Sperm count (million/mL)	33.45	27.78	0.01	110.00
Progressive motility (%)	16.89	14.47	0.00	60.00
Non progressive motility (%)	17.59	13.49	0.00	48.00
Immotile (%)	58.11	27.43	0.01	100.00
Morphology (%)	7.11	7.01	1.00	25.00
Right transverse epididymal diameter (mm)	7.54	1.99	4.40	11.70
Left transverse epididymal diameter (mm)	7.51	1.67	5.30	11.50
Left Varicocele Lying (mm)	3.40	0.86	2.40	4.60
Left Varicocele Standing (mm)	3.74	0.95	2.50	5.80
Follicle Stimulating Hormone (mIU/ml)	6.44	4.23	2.5	21
Right longitudinal diameter of testes (mm)	41.15	3.90	32.00	48.00
Right transverse diameter of testes (mm)	21.00	2.66	17.00	28.00
Left longitudinal diameter of testes (mm)	40.11	3.89	32.00	48.00
Left transverse diameter of testes (mm)	20.33	2.80	16.00	27.00
Left testicular volume (mL)	8.9	2.96	4.93	14.41
Right PSV of sub capsular artery (cm/sec)	6.35	1.95	3.43	11.39
Left PSV of sub capsular artery (cm/sec)	6.36	1.28	4.22	9.50
Right PSV of intra parenchymal artery (cm/sec)	4.95	1.03	3.43	8.20
Left PSV of intra parenchymal artery (cm/sec)	5.46	1.67	3.37	11.71
Right RI of sub capsular artery	0.75	1.07	.35	6.10
Left I RI of sub capsular artery	0.54	0.08	.32	.70
Right RI of intra parenchymal artery	0.52	0.07	.40	.67
Left RI of intra parenchymal artery	0.49	0.14	.26	1.00

*PSV* peak systolic velocity; *RI* resistive index

**Table 2:** Correlation coefficient (r) between semen quality parameters and testicular blood flow in varicocele patients (n = 27)

Variable	Sub capsular artery				Intra parenchymal artery			
	Peak systolic velocity		Resistive index		Peak systolic velocity		Resistive index	
	r	*p*-value	r	*p*-value	R	*p*-value	r	*p*-value
Sperm count (million/mL)	0.329	0.094	−0.194	0.332	0.001	0.998	−0.76	0.012
Progressive motility (%)	0.094	0.640	−0.505	0.007	− 0.160	0.424	− 0.498	0.008
Non-progressive motility (%)	0.209	0.296	−0.194	0.333	−0.098	0.626	−0.325	0.098
Immotile (%)	0.220	0.270	0.291	0.141	0.226	0.258	0.206	0.304
Morphology (%)	0.463	−0.015	−0.157	0.43	0.14	0.48	−0.152	0.45

### VBGs analysis

Table 3 summarizes the comparison of various parameters of VBGs analysis of varicocele veins (internal and external spermatic) with the peripheral vein. Generally, the changes in VBG determinants were more pronounced in the internal spermatic vein than in the peripheral vein. The pH was higher (*p < 0.01*) in the internal spermatic vein compared with the external spermatic and the peripheral veins. The pO_2_ and sO_2_ were elevated (*p* < 0.01) in the internal spermatic vein compared with the peripheral vein. Serum HCO_3_ concentration was lower (*p* < 0.01) in both internal and external spermatic veins compared with the peripheral vein. The pCO_2_ was also lower (*p < 0.01*) in both varicocele veins compared with the peripheral veins (Table 3). There was no significant correlation between various parameters of VBGs of both internal and external spermatic varicocele veins with their respective testicular blood flow, semen quality parameters and the diameter of varicocele veins and testicular volume (data not shown).

**Table 3:** Comparison of venous blood gas analysis of varicocele veins with peripheral vein

Items	Veins			*p*-value
	Internal spermatic *n* = 27	External spermatic *n* = 9	Peripheral *n* = 22	
pH	7.38 ± 0.04^a^	7.34 ± 0.05^b^	7.34 ± 0.04^b^	0.004
pO_2_ (mm of Hg)	86.36 ± 40.47^a^	49.98 ± 7.45^b^	52.64 ± 34.01^b^	0.002
sO_2_ (%)	91.02 ± 7.18^a^	81.24 ± 7.30^ab^	71.06 ± 18.60^b^	< 0.001
pCO_2_ (mm of Hg)	36.87 ± 7.02^b^	40.03 ± 6.58^b^	46.47 ± 7.55^a^	< 0.001
HCO_3_ (mEq/L)	21.4 ± 3.53^b^	21.23 ± 2.91^b^	24.60 ± 3.19^a^	0.003

Values (mean ± SD) with different superscripts in a same row differ significantly. p and s stand for partial pressure and saturation respectively. n shows the number of blood samples analyzed

## Discussion

The current study demonstrates unique characteristics of the VBGs of internal spermatic varicocele veins, which are different from the peripheral veins. The internal spermatic veins have higher pH, sO_2_, and pO_2_ levels but lower HCO_3_ and pCO_2_ levels in comparison to the peripheral vein. On the other hand, the external spermatic varicocele veins revealed different VBGs profile that was similar to the peripheral veins, except lower HCO_3_ and pCO_2_ levels. Previously, between 1968 and 1989, there were only three studies that addressed the composition of venous blood in varicocele veins [14–16]. However, there were conflicting results. Today, we have better methods of measuring blood gas levels and a better opportunity of measuring VBGs during the microsurgical varicocelectomy. This procedure is performed under microsurgical magnification and allows isolation of individual groups of veins thus providing better opportunity of precise blood sampling from different groups of varicocele veins before their ligation. In current study, the VBGs analysis was determined from venous blood, drawn individually from the internal spermatic varicocele veins, the external spermatic varicocele veins, and the peripheral vein. A single surgeon, who is also the principal investigator, performed all the procedures and was blinded to the results until the end of study.

Our results have shown a clear shift of VBGs profile of the internal spermatic veins toward an arterial pattern. The exact mechanism is not clear, but renospermatic backflow, low oxygen consumption due to lower testicular function, or the presence of arterio-venous communications may be a possible clue to this change. There are a few human and multiple animal studies that demonstrate the presence of an arterio-venous shunt in the spermatic cord and testes [8–12]. Based on the available anatomical findings reported in literature [8–12], we suspected that varicocele veins might have different venous composition of blood gases than the classical VBG levels. At the completion of study, it was confirmed that sO_2_ and pO_2_ levels were significantly higher in the internal spermatic varicocele vein compared with the peripheral vein. Nevertheless, further anatomical documentation of an arterial venous shunt mechanism is still required. Donhue and Brown reported in 1969 that the internal spermatic veins of varicocele patients had higher oxygen tension when the blood was drawn from the vein by inserting cannula in the direction of kidney. Anyhow this was not seen when the cannula was directed towards the testes. The authors proposed that the reflux of renal blood might be responsible for this mechanism [15]. In our experience, although we have drawn blood by inserting cannula towards the direction of testes, still we have observed higher oxygen tension in venous blood of internal spermatic veins. In contrast to our findings, Yan reported in 1989 that oxygen saturation was lower in varicocele veins, and suggested that hypoxemia and metabolic acidosis affected spermatogenic function [16]. There is a possibility that the authors might have taken samples from the most accessible external spermatic veins. These veins exhibit lesser increase in intravenous pressure on valsalva compared to internal spermatic veins [22] and have oxygen tension similar to the peripheral veins.

In order to understand the significance of these findings, we determined the correlation of VBGs with testicular blood flow and semen parameters but no significant correlation was found.

## Conclusion

Internal spermatic varicocele veins have significantly higher oxygen content and pH but lower HCO_3_ and pCO_2_ compared with the peripheral veins. The clinical importance of VBGs is difficult to ignore and the above-mentioned variation in blood gases may be a missing link or this may be another possible cause of higher intravenous pressure in these veins, to understand the pathophysiology of varicocele [22]. Further investigations are required to determine the significance of these findings.

## Abbreviations

CDUS: Scrotal Color Doppler ultrasonography
HCO_3_: Bicarbonate
pCO_2_: Partial Pressure of Carbon dioxide
pO_2_: Partial Pressure of Oxygen
PSVs: Peak Systolic Velocities
RIs: Resistive Indices
sO_2_: Oxygen Saturation
VBGs: Venous Blood Gases
